# I am all ears: Maximize maize doubled haploid success by promoting axillary branch elongation

**DOI:** 10.1002/pld3.226

**Published:** 2020-05-15

**Authors:** Miin‐Feng Wu, Alexander Goldshmidt, Daniel Ovadya, Huachun Larue

**Affiliations:** ^1^ Bayer U.S. ‐ Crop Science Chesterfield MO USA; ^2^ Bayer U.S. ‐ Crop Science Woodland CA USA; ^3^ Present address: Department of Field Crops Science Institute of Plant Science Agricultural Research Organization The Volcani Center Rishon Lezion Israel

**Keywords:** axillary branches, colchicine, doubled haploid, maize, plant growth regulator

## Abstract

The maize doubled haploid (DH) technology plays an important role in accelerating breeding genetic gain. One major challenge in fully leveraging the potential of DH technology to accelerate genetic gain is obtaining a consistent seed return from haploid (DH0) plants after chromosome doubling. Here we demonstrated that DH0 seed production can be increased by increasing the number of mature axillary female inflorescences (ears) at anthesis. To determine the maximum capacity of a maize plant to develop ears, we first characterized the developmental progression of every axillary meristem. We found that all axillary meristems developed to a similar developmental stage before the reproductive transition of the shoot apical meristem (SAM). Upon reproductive transition of the SAM, all axillary meristems are released for reproductive development into ears in a developmental gradient reflective on their positions along the main stem. However, under most circumstances only the top one or two ears can generate silks at anthesis. We found that applying the GA inhibitor paclobutrazol (PAC) during the early reproductive transition of axillary meristems increased the number of silking ears at anthesis, leading to increased success of self‐pollination and seed production. These results provide a blueprint to improve DH efficiency and demonstrate the potential of breeding innovation through understanding crops’ developmental processes.

## 1 INTRODUCTION

Commercial maize breeding has seen significant improvement in yield over the past few decades (Butruille et al., 2015). Biotechnology traits and agronomic practices are undoubtedly contributing factors, but the main driving force for yield increase is the genetic gain from plant breeding (Duvick, 2005). The hybrid nature of commercial maize seed dictates a two‐step process in the breeding workflow: inbred‐line development and hybrid testing (Duvick, 2001; Duvick & Cassman, 1999). Modern molecular and predictive analytical technologies, such as marker‐assisted selection (MAS), genotyping‐by‐sequencing (GBS), and genome wide selection (GWS) have increased the breeding capacity exponentially in selecting inbred lines with high yield potential. In addition, incorporating novel breeding methods such as doubled haploid (DH) significantly reduces cycle time, leading to higher genetic gains. Unlike the traditional workflow in which multi‐generational self‐pollination is required to generate near isogenic lines, DH produces homozygous inbred lines in only two generations (Butruille et al., 2015). The DH process in maize breeding involves: (1) creating developmental crosses (F1); (2) in vivo induction of haploid from F1; (3) haploid selection and (4) chromosome doubling of the selected haploids. In the commercial seed industry, steps (1) and (2) are performed in a large‐scale field setting, whereas step (3) has been achieved with the combination of a high oil marker in the haploid inducer genome and automated kernel sorting on the basis of kernel oil content in a high throughput manner (Butruille et al., 2015; Li, Xu, Jin, & Chen, 2009). Haploid plants with 1n chromosomes are sterile, and chromosome doubling to generate 2n chromosomes is necessary for fertile gametes and ultimately seed production. This chromosome doubling step is currently a bottleneck in the DH process. In general, we observed that more than half of DH0 plants produce less than 50 seeds. This insufficient number of seeds requires an additional growth cycle to obtain enough seeds for the following hybrid testing steps, which limits the potential of DH technology in driving genetic gain.

The microtubule disrupting compound colchicine is commonly used to duplicate the chromosomes of haploid plants. Colchicine inhibits mitotic spindle formation in the cell cycle M phase, implying colchicine acts most efficiently on dividing cells. In current practice, colchicine is delivered to seedlings targeting the shoot apical meristem (SAM) as it is the source of progenitor cells for reproductive organs (Prigge & Melchinger, 2012). However, at a given time window only a small percentage of cells at the SAM periphery are undergoing the M phase (Reddy, Heisler, Ehrhardt, & Meyerowitz, 2004), causing stochastic and unpredictable chromosome duplication in SAM cells after colchicine treatments. As a result, colchicine‐treated haploid plants (DH0) are chimeric in ploidy levels, often with partially fertile ears or tassels. We often observed that many secondary, tertiary or even tiller ears from colchicine‐treated DH0 plants produced silks after anthesis and could be pollinated by donor pollens to set seeds, indicating that their chromosomes were duplicated. However, due to these ears not producing silks at anthesis and thus not available for self‐pollination, we are unable to fully leverage the value of these fertile ears. In addition, we frequently observed that a partially fertile DH0 tassel could still generate sufficient pollen to pollinate many ears. We, therefore, reasoned that increasing the number of silking ears at anthesis should in turn increase the chance for optimal DH seed return.

In most modern maize lines, only the topmost ear (the primary ear) produces silks at anthesis. To solve this problem, our objective is to (1) uncover the maximum number of ears a single modern commercial maize inbred line can generate by studying the developmental progression of all axillary meristems, and (2) identify ways to increase the number of silking ears at anthesis. Here, we demonstrated the possibility of producing multiple seed‐bearing ears (productive ears) on a single maize plant, resulting in a significant increase of overall seed return from DH0 plants.

## 2 ALL MAIZE AXILLARY BRANCHES HAVE THE CAPACITY TO DEVELOP INTO EARS

**FIGURE 1:**
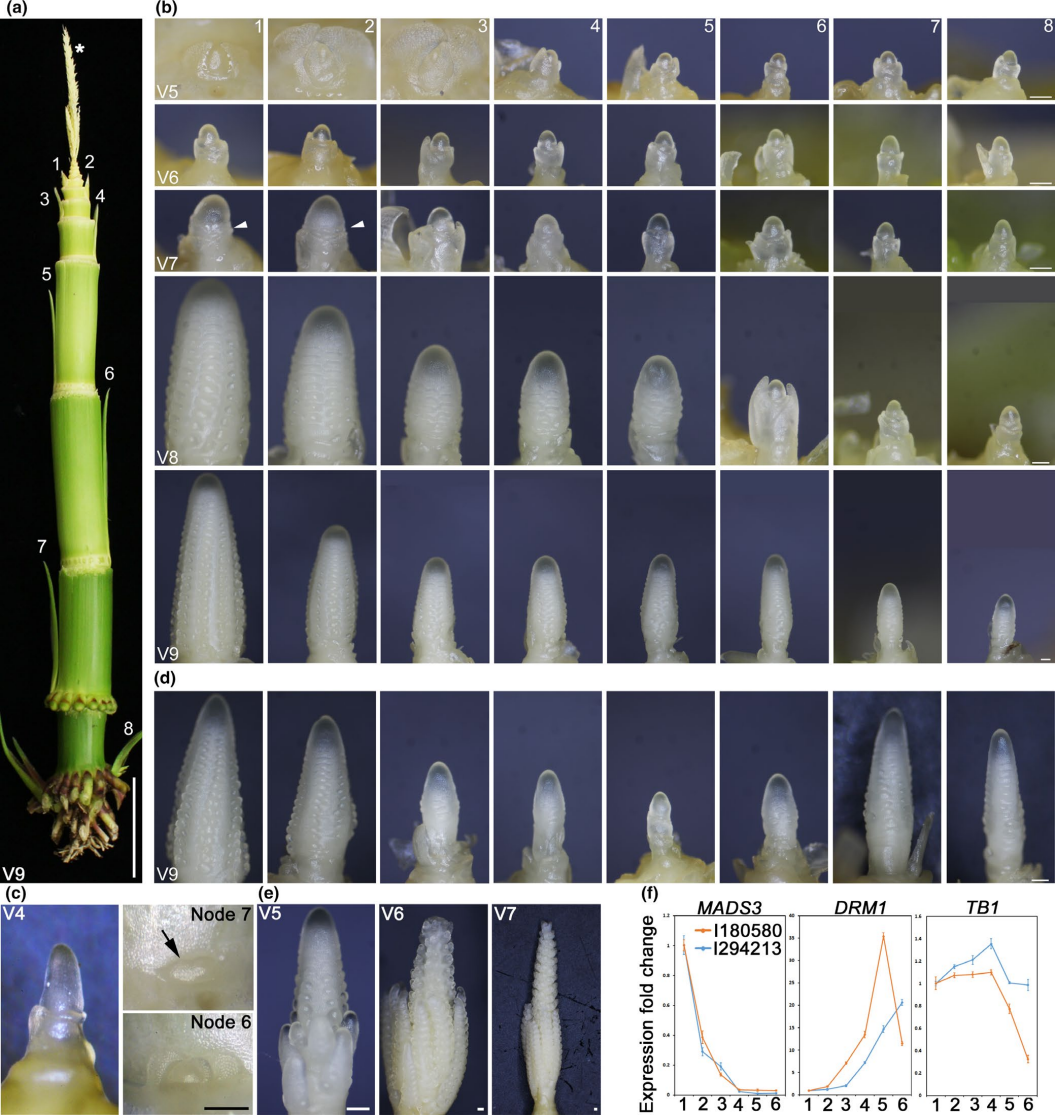
All maize axillary meristems can develop into ears. (a) A V9 inbred line I294213 with all leaves removed to reveal axillary buds. Numbers indicate the position of axillary bud with 1 being the youngest and topmost bud. Asterisk: tassel. Scale bar: 5 cm. (b) Inbred line I294213 ear development from different V stages. Morphological changes indicating reproductive development occurs at V7 in the top two ears. Arrowhead: spikelet pair meristem. Scale bar: 0.2 mm. (c) V4 I294213 shoot apical meristem and the top two visible axillary meristems. Axillary buds that will become ear1 to 3 are not visible at this stage. Arrow: Initiated axillary meristem. Scale bars: 0.2 mm. (d) Prolific inbred line I180580 ears at V9. Development of the lowest three ears (6, 7 and 8) are more advanced than the middle ears (3 to 5). (e) V5 to V7 tassels of inbred line I294213. (f) *MADS3*, *DRM1,* and *TB1* expression from ears 1 to 6 of V6 Inbred line I294213 and I180580. Numbers in the X‐axis indicates the ear position as in (b). Expression levels are normalized to ear1. Shown are mean ± *SEM* of three replicates

**FIGURE 2:**
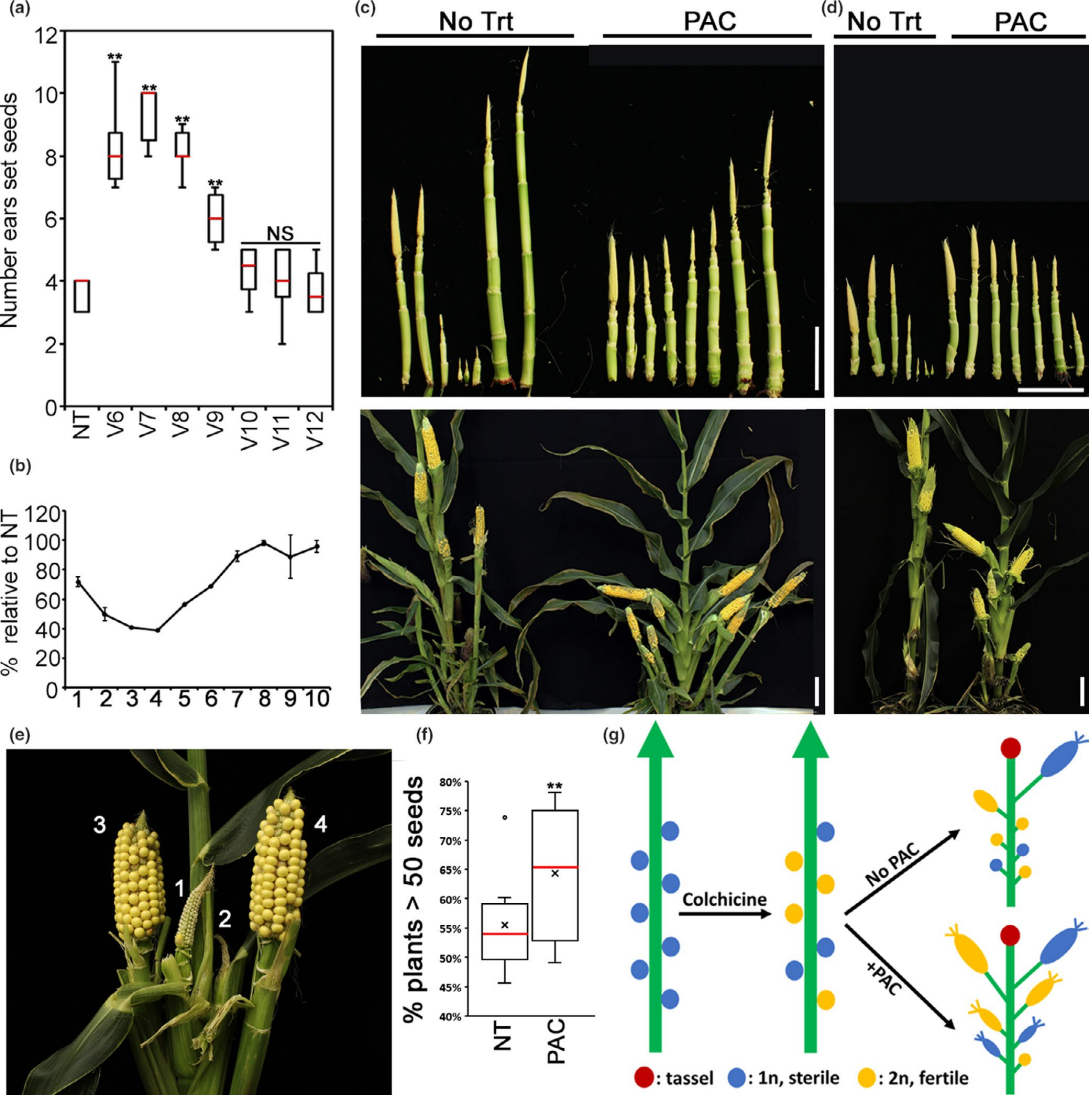
Paclobutrazol treatments increase number of ears setting seeds. (a) PAC treatment at V6‐V7 has the highest number of ears producing seeds. The ear number decreases with the later developmental stage treatments. Shown here are results from inbred line I180580. Red bars: median. *n* = 5–8. **: *p* < .05. p‐value: pairwise *t* test. NT: no treatment. NS: not significant. (b) PAC treatments cause reduced internode elongation. Numbers in the X‐axis indicate the node number with 1 being the first node above the soil surface. Internode length of PAC‐treated plants are normalized over those of untreated plants. NT: no treatment. Shown are results from inbred line I294213 with mean ± *SEM* of two replicates. (c, d) Effects of PAC treatment on inbred lines I180580 (c) and I294213 (d). Treatments in (c) and (d) occurred at V7. Upper panels: ear shoots at anthesis showing elongated shanks after PAC treatments. Lower panels: Plants from 2 weeks after pollination showing increased number of ears with seeds. Scale bars: 10 cm. (e) PAC treated DH0 plants from a segregation population of maize breeding materials. Ear1 and 2 of the plant shown here are sterile, whereas ear3 and 4 are completely fertile. (f) PAC treatment increases percentage of DH0 plants with more than 50 seeds. Red bars: median. X: mean. *n* = 9. **: *p* < .05. p‐value: pairwise *t* test. (g) Diagram showing how PAC treatments maximize DH seed production. Effects of colchicine on chromosome duplication is stochastic depending on the cell division status at the time of treatment. A possible scenario is that the primary ear (ear1) might be sterile (1n) while lower ears could be fertile (2n). PAC treatments promote axillary branch elongation of lower ears, increasing the number of silking ears at anthesis and the chances of successful self‐pollination that gives rise to productive ears with seeds

The maize plant architecture underwent dramatic changes from its predecessor teosinte (Hake & Ross‐Ibarra, 2015). Increased apical dominance has been selected through domestication to enable better harvestability (Doebley, Stec, & Hubbard, 1997; Duvick, 2005; Wills et al., 2013). Unlike teosinte, which has multiple elongated branches topped with a male inflorescence (tassel), most modern maize lines have only one or two short branches with a seed‐bearing female inflorescence (ear) at maturity (Doebley et al., 1997). Since seed quantity plays an important role in the maize breeding workflow, having the ability to increase the number of seed‐bearing ears will add tremendous values to the breeding pipeline. To uncover the maximum number of ears a maize plant is capable of generating, we tracked the axillary meristem reproductive development in a B73‐derived commercial elite inbred line I294213. Ears arise from the axillary buds at the axil of the leaf (Figure 1a). The axillary buds are initiated acropetally until reproductive transition of the SAM into a tassel (Figure 1c,e). The axillary meristem is first initiated as a dome shaped structure at the leaf axil followed by initiation of the prophyll and husk leaf primordia (Figure 1c; Lejeune & Bernier, 1996). The growing prophyll and husk leaves eventually envelope the axillary meristem to form an axillary bud (Figure 1a–c). The initiation of axillary meristems stops at the time of SAM reproductive transition. After the tassel starts to differentiate (Figure 1e), the younger axillary meristems continue to develop until all axillary meristems reach the same developmental stage (Figure 1b, V5 vs. V6). Despite being morphologically similar to the lower axillary meristems at this stage, the topmost axillary meristem that will become the primary ear has significantly higher mRNA expression of a floral identity APETALA 1 class MADS box transcription factor, *MADS3* (Heuer et al., 2001; Figure 1f), indicating this meristem has started transitioning to reproductive development. Interestingly, the expression levels of teosinte branch 1 (*TB1*) stays constant across all meristems, whereas the bud dormancy marker gene *DRM1* (Stafstrom, Ripley, Devitt, & Drake, 1998) displays higher expression in lower axillary meristems (Figure 1f). *TB1* is a domestication gene whose higher expression in maize suppresses branching (Doebley et al., 1997). These results suggest that the suppression of lower ear outgrowth in inbred line I294213 might be via a differentially expressed regulator independent of *TB1*. Starting at V7, the bract primordium and spikelet pair meristem has become visible in the top two meristems, whereas the lower meristems stay morphologically indistinguishable from earlier plant growth stages (Figure 1b). At V8, the top two meristems continue to elongate and initiate more spikelet pair meristems (Figure 1b). Very interestingly, despite the initial pause, the lower axillary meristems commence reproductive development into ears in a basipetal order, forming a developmental gradient reflective on the ear position (Figure 1b). In a prolific inbred line I180580, we observed similar ear developmental patterns at V6, consistent with the *MADS3* expression patterns. However, the expression of *DRM1* and *TB1* in I180580 was significantly different from I294213 in ear 6, the lowest ear analyzed. The expression level of *DRM1* in I180580 gradually increased until ear 5 and showed a significant reduction in ear 6 (Figure 1f). Similarly, *TB1* expression decreased in ear 6 (Figure 1f). The change in *DRM1* and *TB1* expression is reflective on the ear development as the lower ears in I180580 are more developmentally advanced at a later stage (V9; Figure 1d). These lower ears eventually become elongated branches at maturity (Figure 2c). The relative low expression of *MADS3* in V6 I180580 lower ears further suggests that the release of dormancy may proceed reproductive transition.

The eventual transition of all maize axillary meristems to reproductive development demonstrates that maize axillary meristems do not stay dormant throughout their life cycles (Figure 1b). Our results show that all axillary meristems reach a morphologically similar developmental stage around V6. Instead of being dormant permanently, the lower meristems are poised to be released for differentiation once the top meristem transitions to reproductive development. This further shows that all maize axillary meristems have the potential to form fully productive ears.

## 3 DEVELOPMENTAL STAGE‐SPECIFIC GA INHIBITOR TREATMENT PROMOTES MAIZE AXILLARY BRANCH ELONGATION AND SYNCHRONIZES SILKING AT ANTHESIS

The finding that all axillary meristems can differentiate into ears prompted us to ask whether the axillary branch elongation could be manipulated to enhance the number of silking ears that can be self‐pollinated at anthesis. Complex interactions between the environment and hormones play a central role in branch growth and patterning, a main determinant for the overall plant architecture (Wang, Smith, & Li, 2018). Any given plant can show dramatically different architectures under different environmental parameters, such as growth density, light quality and soil nutrient profiles, suggesting plant architecture is plastic and could potentially be manipulated. Commercially mass‐produced plant growth regulators (PGR) present a viable option for modifying plant architecture without pertinent genetic modifications (Rademacher, 2000, 2016). For example, applying PGR has been a common practice in the horticulture industry to achieve market‐appealing plant architectures. Among a plethora of commercially available PGRs, it was previously shown that gibberellin (GA) biosynthetic inhibitors promoted ear shoot elongation and internode length reduction (Xu, York, Miller, & Cheikh, 2004). To understand whether GA inhibitors play a role in promoting maize axillary branch elongation and ultimately increasing the seed production, we tested the GA inhibitor paclobutrazol (brand name PACZOL, PAC) on commercial elite inbred lines I294213 and I180580. We found that PAC treatments increased the number of ears setting seed in both inbred lines tested (Figure 2a,c,d) while reduced internode length as previously described (Figure 2b; Xu et al., 2004). Most strikingly, we also observed that every single axillary meristem from the main shoot had the potential to form a productive ear. We obtained the highest number of productive ears when plants were treated at V6–V7, around the time of the primary ear reproductive transition (Figures 1b and 2a). Since all the ears were derived from manual self pollination at anthesis to mimic the inbred creation process in maize breeding, our results further indicate that every single ear generates silks synchronously at anthesis with PAC treatment around V6–V7. When plants were treated at earlier growth stages, we did not observe increased ear number on the main stem, whereas branch elongation from the basal nodes was increased (data not shown). Based on these results, we conclude that the best treatment timing to obtain the maximum number of silking ears at anthesis is at the very beginning of the primary ear reproductive transition. Since the developmental status of all ears is similar at this timepoint, we posit that PAC treatments promote a simultaneous release of multiple axillary branches for elongation. We saw similar responses in lines of different prolificacy under the same growth conditions and density (Figure 2c,d), suggesting a high penetrance of phenotypes triggered by PAC treatments.

## 4 POSSIBLE MECHANISMS OF PAC IN PROMOTING BRANCHING

In the classic theory of branching regulation, the apical derived plant hormone auxin inhibits branch activation and the branch is activated once the auxin source is removed (Thimann & Skoog, 1933). This observation is later explained by the auxin canalization model. In this model, auxin polarizes and amplifies its own transport by upregulating export carriers to form a basipetal auxin “canal” in the main stem (Bennett, Hines, & Leyser, 2014; Sachs, 1975; Sauer et al., 2006). The dominant auxin stream in the main stem constitutes a weak sink that prevents other sources of auxin feeding into the stream (Domagalska & Leyser, 2011). Since auxin export is necessary for a sustained branch elongation, this inhibition thus prevents axillary branch outgrowth (Brewer, Dun, Ferguson, Rameau, & Beveridge, 2009; Chabikwa, Brewer, & Beveridge, 2019; Reinhardt et al., 2003). The auxin source in the main stem comes from the shoot apex and young leaves (Ljung, Bhalerao, & Sandberg, 2001). To activate axillary buds, the apical auxin source needs to be removed either by decapitating the shoot apex or when the meristem is transitioned to reproductive development (Napoli, Beveridge, & Snowden, 1999). As a result, auxin flow in the main stem is reduced, allowing auxin export from the axillary bud and the subsequent activation of the bud. The order of bud activation is determined by the distance from the original auxin source. The topmost bud that is closest to the apex is activated first once the apical auxin source is removed (Prusinkiewicz et al., 2009). Auxin export from the topmost branch in turn inhibits the export from the lower branches until reproductive transition (Balla et al., 2016; Prusinkiewicz et al., 2009).

Based on our findings and the current theory of branch activation, we propose a model extrapolated from the auxin canalization theory to explain the patterns of maize axillary branch activation. Like in the auxin canalization theory, the apical supply of auxin weakens as the SAM transitions to reproductive development. This weakened supply of apical auxin allows the auxin flow from the axillary bud feeding into the main stem, resulting in branch outgrowth and subsequent reproductive development into ears. Similarly, the timing of these developmental changes is dependent on each axillary meristem's distance from the diminishing apical auxin source. This might explain why the ear developmental progression forms a gradient reflective on each ear's position along the main stem. In some pedigrees, the auxin flow may be decayed toward the base of the plant and insufficient to inhibit basal branch activation, resulting in more advanced ear development in the basal nodes (Prusinkiewicz et al., 2009; Figure 1d). The higher frequency of basal branch elongation in some pedigrees suggests a plausible genetic contribution in maintaining the auxin transport stream.

According to our model, it is plausible that the PAC treatments may influence auxin flow in the stem to promote branching. Research has shown that polar auxin transport in the inflorescence is reduced in Arabidopsis GA synthesis and signaling mutants (Willige, Isono, Richter, Zourelidou, & Schwechheimer, 2011). Apical derived auxin from the shoot promotes root elongation by enhancing the GA‐mediated destabilization of the DELLA proteins (Fu & Harberd, 2003). Furthermore, polar localization of the auxin efflux carrier PIN protein in the plasma membrane requires GA signaling (Fu & Harberd, 2003; Lofke et al., 2013). When GA biosynthetic inhibitors are applied to the root, PIN protein is removed from the cell membrane, resulting in loss of auxin transport (Lofke et al., 2013). Another theory is that inhibition of GA signaling induces cytokinin responses which in turn activates transcription of the auxin signaling inhibitor *SHY2/IAA3*, inhibiting PIN1 expression (Moubayidin et al., 2010). Based on these published results, GA inhibitors may play a role in weakening auxin transport in the main stem, mimicking decapitation or shoot reproductive transition.

In addition to auxin transport, several recent studies have shown that sugar acts as the earliest signal for branch activation (Chabikwa et al., 2019; Fichtner et al., 2017; Mason, Ross, Babst, Wienclaw, & Beveridge, 2014). It is hypothesized that a rapidly elongating stem is a strong sink preventing sugar from becoming available to the axillary bud, which then prevents bud activation, whereas a shortened internode constitutes a weaker sink thus allowing for sugar flow into the axillary bud to promote growth (Kebrom, 2017). One major effect of PAC in addition to promoting branching is shortened internode elongation (Best, Johal, & Dilkes, 2017; Xu et al., 2004). The shortened internodes in PAC‐treated plants could potentially be another contributing mechanism of how PAC promotes branching. Although increased branching is often associated with mutants of shorter stature (Kebrom, 2017), one exception is the maize *brachytic2* (*br2*) mutant (Multani et al., 2003; Pilu et al., 2007; Xing et al., 2015). BR2 encodes an adenosine triphosphate (ATP)‐binding cassette transporters of the multidrug resistant (MDR) class of P glycoproteins (PGPs) that is expressed in the internode intercalary meristem and has been shown to play a role in long distance polar auxin transport (Multani et al., 2003). *Br2* mutant is dwarf with reduced capability to transport auxin across the internode (Knoller, Blakeslee, Richards, Peer, & Murphy, 2010). However, increased branching has never been reported in *br2* mutants. This might suggest that increased branching linked to shortened internodes must occur simultaneously with functional polar auxin transport.

## 5 LEVERAGING GA INHIBITOR ACTIVITY TO INCREASE SEED PRODUCTION FOR DH0 PLANTS

Maize is monoecious with female (ear) and male (tassel) reproductive organs in separate positions on the same plant. Since the goal of the DH process is to create homozygous inbred lines, chromosomes in both tassel and ears must be duplicated to allow formation of fertile gametes for successful self‐pollination. To maximize the success of the DH process, we tested the unique effect of PAC in promoting the number of silking ears at anthesis to increase DH seed return. Figure 2e shows an example of how PAC treatment benefits the DH process. Because of the stochastic nature of colchicine treatment, the primary ear (ear1) is not necessarily completely fertile, whereas lower ears could be fully fertile depending on the cell division status at the time of colchicine treatment (Figure 2g). With PAC treatment, lower ears produce silk at anthesis and can be self‐pollinated. This leads to a significant increase in overall seed production when the lower ears are fertile (Figure 2f).

## 6 FACTORS IMPORTANT FOR CONSISTENT RESPONSE

One main variable to consider in applying plant growth regulators by root drenching is that the efficacy can be influenced by the components in soilless potting medium. For example, the calcine clay‐based particle, Turface, significantly reduces triazole‐type growth regulator's efficacy via hydrophobic interactions with the growth regulators (Best et al., 2014). Pine bark can reduce efficacy depending on the degree of composting (Million et al., 1998). It is thus imperative to standardize the potting medium for a consistent response.

In addition, due to the combination of shorter internodes from PAC treatment and the overall shorter stature of DH0 plants, treatments often need to occur at a slightly later timepoint than diploid plants to prevent over‐stunting. However, the treatment still needs to occur within the developmental window when branch development is plastic and before the dominance of the primary ear is fully established. The ideal treatment timepoint for DH0 plants, therefore, should occur no later than 2 weeks after primary ear reproductive transition.

Another important factor to consider for a consistent response is plant density. We have consistently observed in the controlled environment that PAC losses its efficacy when plants are kept at a very high density. This suggests that the shade avoidance response triggered by high plant density might override the branch promoting effects of PAC.

## 7 CONCLUSIONS

Our results show that maize axillary meristems in the leaf axils are first initiated as a vegetative structure. The initiated meristem stays poised until the SAM undergoes reproductive transition into the tassel. Soon after the tassel starts to differentiate, the axillary meristem resumes growth and transitions to reproductive development from the topmost meristem then progressing in a basipetal order. Instead of staying dormant, all maize axillary meristems have the capacity to differentiate into ears. This feature provides an opportunity of manipulating axillary branch elongation to increase seed production for breeding purposes. We found that the maize axillary branch elongation can be promoted by the GA inhibitor paclobutrazol (PAC), and treatments with PAC around primary ear reproductive transition result in the highest number of productive ears. This is especially beneficial for the incompletely fertile DH0 plants as it increases the chances of successful self‐pollination and seed production.

Domestication of maize selected for a less branched plant architecture, making it a very simple and visual model for further understanding of branching regulation. Research on maize axillary branch development will contribute to valuable impact on agriculture. Future directions would include (1) establishing a direct connection between the auxin canalization theory and maize axillary meristem development, and (2) dissecting other signaling pathways and regulatory mechanisms controlling the dormancy release of axillary meristems. Deepened understanding in the development of maize axillary meristem will pave the road for further germplasm enhancement that is more adaptive to different environments.

## 8 MATERIALS AND METHODS

### 8.1 Plant materials and growth conditions

Elite inbred lines I180580 (US patent 7518043) and I294213 (US patent 7166779) were used for phenotypic and gene expression analyses. Inbred lines I180580, I294213 and segregating DH0 populations were used for paclobutrazol treatments. Plants were grown in Redi‐Earth growth medium (SunGro Horticulture) in growth chambers set to 14‐hr light/10‐hr dark. The growth chambers contained 1:1 ratio of high pressure sodium and metal halide bulbs with the light intensity set to 650 µmol m^−2^ s^−1^. Daytime temperature was set 28°C and night time temperature was set to 20°C.

### 8.2 Paclobutrazol treatments

Inbred lines were treated with 60 ml of 2.5% PACZOL (OHP Inc.) containing 6 mg of active ingredient paclobutrazol. PACZOL solution was injected into the root zone near the base of the stalk using serological pipets. DH0 plants were treated with the same concentration of PACZOL on the 28th day after colchicine treatment, followed by another treatment 7 days later.

### 8.3 Quantitative RT‐PCR

Total RNA was extracted by TRIzol Reagent (Thermo Fisher Scientific). About 500 ng RNA was used for reverse transcription using SuperScript IV first strand synthesis kit (Thermo Fisher Scientific). Real‐time PCR was performed using Power SYBR PCR master mix (Thermo Fisher Scientific). Relative expression was calculated from threshold cycle values and normalized to those of Zea mays eukaryotic translation initiation factor 1A (EIF1A). Expression fold change was calculated by normalizing to the relative expression levels of ear 1. Primers used: EIF1A‐F gtggaaagaacgaggctgac; EIF1A‐R ccacaccttcttgtgcatct; MADS3‐F cggcagcaaactaagctaaaa; MADS3‐R caaaagcaccgctagattca; DRM1‐F gagagggctggacgaagaa; DRM1‐R gatcaccacccagtcgtaca; TB1‐F tcctcaacacgtccaagtcc; TB1‐R gggcttctgatctcctcctc.

## AUTHORS’ CONTRIBUTIONS

H. L. initiated the project. H. L. and M.‐F. W. designed experiments. M.‐F. W. performed experiments, collected and analyzed data. D. O. and A. G. provided preliminary results and comments to the manuscript. M.‐F. W. and H. L. wrote the manuscript.

## Supporting information

Supinfo
