# Supplementary material for: I am all ears: Maximize maize doubled haploid success by promoting axillary branch elongation
Source: Plant Direct. 2020 May 15;4(5):e00226. doi: 10.1002/pld3.226 (PMC7227119; doi:10.1002/pld3.226)
Supplement: Supplementary file 1 — Supinfo [file PLD3-4-e00226-s001.pdf]

Dear Editors and Reviewers,

We thank the reviews very much for the comments to help us improve the manuscript. Please see the following for our point-to-point response.

Reviewer #1:

The manuscript by Wu et al. presents a very interesting analysis about the developmental progression of axillary meristems in maize and how manipulate it.

I suggest the following corrections to can have a suitable manuscript for publication.

In the Line 105, the authors should describe the characteristics of the prolific inbred line I180580.

We have modified the sentence in line 105 to clarify the phenotypic description. We have also added the US patent numbers of both inbred lines used in this manuscript to the materials and methods. The patents contain detailed description on the characteristics of the inbred lines.

In my opinion, the authors should show the expression of DRM1, TB1 and MADS3 also in the prolific inbred line I180580, where the expression of MADS3 should be increased in the lowest two ears, and DRM1 should be lower.

We have included qRT-PCR results of DRM1, TB1 and MADS3 in I180580. Although we did not see increased MADS3 expression in the lowest ears we analyzed, we did observe significant reduction of DRM1 expression in these ears.

Do the authors have quantitative data from their treatments with PAC about the effect in shortened internode elongation?

In that case, they should show it to support the statement about PAC is promoting shortening in internode elongation.

We have included quantitative data of internode length reduction after PAC treatments in figure 2B. We have also cited Xu,2004 which showed PAC's effect on internode length reduction.

In the Figures 2A, D and E, the authors should describe what inbred line is it.

We have added description of inbred lines used in figure 2A, B, C, D. The plants used in figure 2E and F were from segregation DH populations of maize breeding materials. Unfortunately, we do not have the exact genetic information on these plants. We have included this information in the figure legends and in materials and methods.

Why the authors are not showing any information about used methods?

Materials and methods are included.

Reviewer #2:

You should reconsider your hypothesis regarding the effect of PAC on branching.

We have modified the description of PAC’s role in branching with more tentative language.

Include a materials and methods section in your MS.

Materials and method are included.

With best regards,

Miin-Feng Wu and Huachun Larue
